# Combined Transfection of the Three Transcriptional Factors, PDX-1, NeuroD1, and MafA, Causes Differentiation of Bone Marrow Mesenchymal Stem Cells into Insulin-Producing Cells

**DOI:** 10.1155/2012/672013

**Published:** 2012-06-19

**Authors:** Guo Qing-Song, Zhu Ming-Yan, Wang Lei, Fan Xiang-Jun, Lu Yu-Hua, Wang Zhi-Wei, Zhu Sha-Jun, Wang Yao, Huang Yan

**Affiliations:** ^1^Department of General Surgery, Affiliated Hospital of Nantong University, Nantong 226001, China; ^2^Department of Surgical Comprehensive Laboratory, Affiliated Hospital of Nantong University, Nantong 226001, China

## Abstract

*Aims*. The goal of cell transcription for treatment of diabetes is to generate surrogate **β**-cells from an appropriate cell line. However, the induced replacement cells have showed less physiological function in producing insulin compared with normal **β**-cells. *Methods*. Here, we report a procedure for induction of insulin-producing cells (IPCs) from bone marrow murine mesenchymal stem cells (BM-mMSCs). These BM-mMSCs have the potential to differentiate into insulin-producing cells when a combination of PDX-1 (pancreatic and duodenal homeobox-1), NeuroD1 (neurogenic differentiation-1), and MafA (V-maf musculoaponeurotic fibrosarcoma oncogene homolog A) genes are transfected into them and expressed in these cells. *Results*. Insulin biosynthesis and secretion were induced in mMSCs into which these three genes have been transfected and expressed. The amount of induced insulin in the mMSCs which have been transfected with the three genes together is significantly higher than in those mMSCs that were only transfected with one or two of these three genes. Transplantation of the transfected cells into mice with streptozotocin-induced diabetes results in insulin expression and the reversal of the glucose challenge. *Conclusions*. These findings suggest major implications for cell replacement strategies in generation of surrogate **β**-cells for the treatment of diabetes.

## 1. Introduction

Type 1 diabetes is characterised by absolute insulin deficiency caused by T-cell-mediated destruction of pancreatic *β*-cells. *β*-Cell replacement is a promising approach for treatment of type 1 diabetes. Islet cell replacement has been considered as the potential cure for diabetes over the past thirty years. However, this treatment is limited by a shortage of pancreas donors and immune rejection against islets. Recently, the methods of obtaining insulin-producing surrogate *β*-Cells from non-*β*-cells through induction or genetic engineering have been investigated, which supports a new sight in Type 1 diabetes treatment [1–4].

Transcription factors control biological processes such as differentiation, proliferation, and apoptosis. They bind to the specific sequence within the region of the promoter or enhancer and activate specialized genes' expression. It has been reported that a number of transcription factors were involved in pancreas *β*-cells' development and function maintaining [5]. It has been reported that PDX-1, NeuroD1 and MafA directly bind to the insulin gene promoter and promote transcription of insulin mRNA and maintenance of *β*-cell function during pancreatic *β*-cell differentiation. Further studies have also shown that the transcription of these three genes and their resultant three protein products are crucial for glucose regulation of insulin production [6]. But whether synergistic effect may induce higher insulin expression and promote non-*β*-cells further differentiated into *β*-cells in vivo or in vitro is still needed to be further studied.

Recently, MSCs were chosen as target cells for transplantation, because of their ability to differentiate into multiple cell types [7, 8], their ability to elude detection by the host's immune system [9], and the relative ease of expanding these cells in cell culture [9]. In our study, a combination of these three transcription factors, which all play a crucial role in glucose induction of insulin gene transcription and pancreatic *β*-cell function, were delivered into mMSCs on adenoviral vectors. After infection, the cells were cultured in defined conditions with epidermal growth factor (EGF) to promote transdifferentiation and were found to have an active endogenous insulin gene. 

There are some findings demonstrating the feasibility of inducing a functional alteration in cultured MSCs by expression of a single master pancreatic regulator gene [10, 11]. In the previous study, we have successfully generated IPCs from bone marrow MSCs by introduction of a human insulin gene [12]. After the transfected cells were injected into the liver of mice with diabetes, we found that the hyperglycemia in mice with diabetes could be reversed effectively [13]. However, the cells for transplantation showed weak glucose responsiveness and immaturity. There remains a sizable gap between induced cells and normal islet *β*-cells. The recent work on induced pluripotent stem cells (iPS cells) and induced neuron formation suggests that a specific combination of multiple transcription factors instead of a single one might be sufficient to directly reprogram adult cells [14, 15]. A number of transcription factors play important roles in the processes of *β*-cell differentiation and to some extent other genes are responsible for maintaining *β*-cell function. Here, in the present study, the combination of PDX-1, NeuroD1, and MafA markedly induces insulin biosynthesis and secretion in mMSCs and thereby this is a novel approach to induce insulin-producing surrogate *β*-cells efficiently for transplantation.

## 2. Materials and Methods

### 2.1. Construction of Recombinant Adenovirus Vectors Harboring Target Gene

The genes of mouse transcription factors PDX-1, NeuroD1, and MafA (gene ID: 18609, 18012, 378435) were obtained by total gene synthesis and gene sequencing to validate that the synthesis was correct. The encoding sequences of PDX-1, NeuroD1, and MafA were amplified and ligated with an internal ribosome entry site sequence-green fluorescent protein (IRES-GFP) by PCR, then cloned into a shuttle vector pDONR221 by BP clonase II enzyme mix (Invitrogen, Carlsbad, CA, USA) according to the manufacturer's protocol. The corrcet recombinant plasmids were then cloned into the pAD/CMV/V5-DEST adenoviral vectors (Invitrogen, Carlsbad, CA, USA) by LR clonase II enzyme mix (Invitrogen, Carlsbad, CA, USA) according to the manufacturer's protocol. Electrophoretic analysis and DNA sequencing were performed to identify the recombinant vectors.

### 2.2. Adenovirus Production

After the cells were counted, the packaging cell line 293A in logarithmic growth phase was incubated in a 6-well culture plate at 37°C, 5% CO_2_ the day before transfection. The sequences of the recombinant adenovirus vectors pAd-Mouse PDX-1-IRES-GFP, pAd-Mouse NeuroD1-IRES-GFP and pAd-Mouse MafA-IRES-GFP were confirmed by gene sequencing and linearized with Pac I and then transfected into the adenovirus packaging cell line 293A using Lipofectamine2000 (Invitrogen, Carlsbad, CA, USA). 48 hours after transfection, cells were detached and transferred to a petri dish. Fresh nutrient medium was added every two or three days. The supernants were collected from 293A cells when most of the cells showed significant cytopathic effect (CPE). Primary adenoviruses were harvested after 3 times of freeze-thawing of the supernants. The primary adenoviruses were used to infect 293A cells in 10 cm petri dishes to make adenoviruses concentrated. Finally the concentrated adenoviruses were stored at −80°C. The control adenovirus expressing green fluorescent protein (Ad-GFP) was prepared as the above-mentioned method. The titer of the adenovirus was determined by an immune method, as follows: the HEK-293 cells that had been infected with adenovirus in different concentrations were reacted with rabbit antiadenovirus polyclonal antibody (1 : 1000) for 1 hour and were then incubated for additional 1 hour with horseradish peroxidase labeled anti-rabbit antibody. After 3,3′-diaminobenzidine (DAB) staining, the titer of the adenovirus was calculated in terms of the number of brown particles formed in different dilutions.

### 2.3. Cell Culture

Bone marrow mMSCs from mice were enriched and expanded in vitro by using the whole bone marrow adherence method according to the previous protocol published from our laboratory [13], with slight modifications [16]. Briefly, two-month-old male C57BL/6J mice were sacrificed and soaked in 75% ethanol for 3 minutes. The femurs and tibiae were dissected away from attached muscle and connective tissues, after which the bones were placed in phosphate buffered saline (PBS) on ice. Either of the ends of each tibia and femur was clipped, and then the bone marrow was extruded by inserting a 21-gauge needle into the shaft of the bone and flushing with rinse solution. Rinse solution consisted of PBS (PH 7.2), 2% fetal bovine serum (FBS; GIBCO BRL, Gaithersburg, MD, USA) and 1 mM ethylene diamine tetraacetic acid (EDTA). Bone marrow cells were collected by centrifugalization and resuspended in Dulbecco's-modified eagle's medium/Ham's Nutrient Mixture F-12 (DMEM/F12; HyClone, Logan, UT, USA) supplemented with 10% FBS, 100 units/mL penicillin, 100 mg/mL streptomycin, 2 mM L-glutamine (Sigma Chemical Company, St. Louis, MO, USA) and 10 ng/mL human basic fibroblast growth factor (bFGF; ProSpec-Tany TechnoGene, Rehovot, Israel) to promote cell proliferation. Cells were plated in 25 cm^2^ culturing flask (Corning Enterprises, Corning, NY, USA) and incubated at 37°C with 5% humidified CO_2_. The nonadherent cells were removed after 72 h, and adherent cells were thoroughly washed twice with PBS. As the cells grew to 80% confluence and were treated with 0.25% trypsin-0.02% EDTA (Sigma Chemical Company, St. Louis, MO, USA) for 5 minutes at 37°C in the ratio 1 : 2 at each passage. Flow cytometry was performed for immunophenotype analysis of mMSCs. mMSCs at passage 3 were trypsinized and washed three times with PBS, then the cells were incubated with the following labeled antibodies: CD14, CD29, CD34, CD44, CD45, and CD105. Labeled cells were analyzed on a FACSort Calibur (BD Biosciences, Franklin Lakes, NJ, USA). To confirm the multipotency character of mMSCs, mMSCs at passage 3 were incubated in osteogenic or adipogenic differentiation medium for 21 days, followed by staining with alkaline phosphatase or oil red, respectively, as described previously [17].

### 2.4. MOI Determination and Cell Infection

Cells were collected from a highly proliferative mMSC culture at passage 3 and plated into 96 well culture plates at the same density. On the next day cells were infected with freshly harvested Ad-GFP at different multiplicity of infection (MOI) from 5 to 5000 in medium containing 2% FBS and incubated for 24 h. Infection efficiency was determined by fluorescence microscope after a further three days, and toxicity was determined by Cell Counting Kit-8 (CCK-8; Dojindo Molecular Technologies, Inc., Kumamoto, Japan). 10 uL of CCK-8 solution was added into every well according to the instructions on the manufacturers' kit, followed by incubation at 37°C with 5% humidified CO_2_ for 2 hours. The absorbance of the infected cells was measured by a microplate reader. The optimal MOI were identified from infection efficiency and toxicity. Ad-Mouse PDX-1-IRES-GFP, Ad-Mouse NeuroD1-IRES-GFP and Ad-Mouse MafA-IRES-GFP were prepared, and then mMSCs were infected with viruses containing the three factors at an optimal MOI. Each of the adenovirus has the same contribution to the optimal MOI, single gene delivery and double infection were also performed. The following day the cells were switched to differentiation medium supplemented with EGF. The infection was repeated in the following days. The cells were infected with Ad-GFP as a control.

### 2.5. Reverse Transcription Polymerase Chain Reaction

Total cellular RNA was isolated using the MicroElute Total RNA Kit (OMEGA BIO-TEK, GA, USA) according to the manufacturer's instructions. After quantification by spectrophotometry, 2 *μ*g of RNA was used for cDNA synthesis with a RevertAid TM First Strand cDNA Synthesis Kit (Fermentas DNA International, Burlington, Canada). Polymerization reactions were performed using a 20 *μ*L reaction volume containing 1 *μ*L of cDNA, with the oligonucleotide primers being as follows: PDX-1 (330bp), 5′-TGAAATCCACCAAAGCTCACGC-3′ (forward primer) and 5′-CCGAGGTCACCGCACAATCT-3′ (reverse primer); NeuroD1(494bp), 5′-GAGGAACACGAGGCAGACAAG-3′ (forward primer) and 5′-AAGAAAGTCCGAGGGTTGAGC-3′ (reverse primer); MafA (402 bp), 5′-CCATCATCACTCTGCCCACCAT-3′ (forward primer) and 5′-CCCGCCAACTTCTCGTATTTCT-3′ (reverse primer); insulin1 (327 bp), 5′-CTATAAAGCTGGTGGGCATCC-3′ (forward primer) and 5′-AACGCCAAGGTCTGAAGGTC-3′ (reverse primer); insulin2 (368 bp), 5′-AGCCTATCTTCCAGGTTATTGTTTC-3′ (forward primer) and 5′-GGTGGGTCTAGTTGCAGTAGTTCTC-3′ (reverse primer); *β*-actin (517 bp), 5′-ATATCGCTGCGCTGGTCGTC-3′ (forward primer) and 5′-AGGATGGCGTGAGGGAGAGC-3′ (reverse primer). Amplification conditions included initial denaturation at 94°C for 10 min, followed by 35 cycles of denaturation at 94°C for 30 sec, annealing at 61°C for 30 sec and extension at 72°C for 30 sec, at last an extension step of 10 min at 72°C.

### 2.6. Immunofluorescence Analysis

After being cultured for 21 days, infected cells were seeded on glass slides in a 12-well culture plates and fixed in 4% paraformaldehyde in PBS. Permeabilizing and blocking was performed in 10% fetal calf serum, 3% bovine serum albumin, and 0.2% triton X-100 in PBS. Then the cells were incubated with primary antibody (rabbit anti-mouse insulin polyclonal antibody 1 : 50; Santa Cruz Biotechnology, CA, USA.) overnight at 4°C. For insulin staining, the cells were further incubated for 2 h at room temperature in the dark, with secondary antibody (Cy3 anti-rabbit 1 : 50; Proteintech Group, Chicago, IL USA). After Hoechst staining for additional 15 min, the slides were washed and examined under the microscope. Images were captured using an Olympus phase contrast fluorescent microscope (Olympus Corporation, Tokyo, Japan).

### 2.7. Insulin Secretion Assay

The infected cells or noninfected cells were preincubated with Krebs-Ringer buffer (KRB) for 1 h, followed by incubation for an additional 1 h in KRB containing 10.0 mM glucose. The buffer was collected and frozen at −80°C until assay. Insulin enzyme-linked immunosorbent assay (ELISA; Cusabio Biotech Co., Wuhan, Hubei, China) was used for the quantitative determination of insulin levels in the collected buffer according to the manufacturer's protocol. All values were determined against a standard curve prepared with mouse insulin.

### 2.8. Establishment of Diabetes Mellitus Models and Cell Transplantation

To set up models of mice with diabetes, adult C57BL/6J mice were injected intraperitoneally with Streptozotocin (STZ; Sigma Chemical Company, St. Louis Missouri, USA) at a dose of 160 mg/kg. Hyperglycemia had been made by the administration of this dose of STZ within 7 days. Blood glucose reached levels >16.7 mmol/L and kept hyperglycemia for 2 weeks at least. Cells for transplantation were prepared at the same time. Transcription factors PDX-1, NeuroD1, and MafA were delivered into mMSCs 3 days before transplantation. Mice were anesthetized with intraperitoneal injection of sodium pentobarbital at 50 mg/kg, followed by the abdominal incision. About 1-2 × 10^6^ infected cells or non-infected cells suspended in 0.2 ml PBS were transplanted into the liver parenchyma of mice with diabetes. For the glucose tolerance test, mice were injected intraperitoneally with 2.0 g of glucose per kg body weight after overnight fast. Blood glucose levels were monitored at the indicated time points (0–120 min) in samples obtained from the tail vein of mice by using One-Touch II portable blood glucose monitor (Lifescan Inc., Milpitas, CA, USA). The mice were sacrificed two weeks after transplantation. To witness the survival of the IPCs and detect the insulin secretion of triple infected cells in the liver tissues, the livers were removed and fixed in 10% formalin. Forty-eight hours later they were cut into serially sections and analyzed by immunohistochemistry. Negative controls were also set up. mMSCs infected with Ad-GFP or mMSCs without any infection were transplanted into the livers as a control. Terminal deoxynucleotidyl transferase-mediated biotinylated-dUTP nick-end labeling (TUNEL) assay was also performed using the In Situ Cell Death Detection Kit, Fluorescence (Roche Applied Science, Mannheim, Germany) to determine whether the injected cells were apoptotic. The livers were taken out immediately for making frozen tissue sections. Frozen tissue sections were rinsed with PBS and treated with 1% Triton X-100 in PBS for 2 min on ice. Slides were rinsed in PBS and incubated for 60 min at 37°C with 50 *μ*L of TUNEL reaction mixture. After washing with PBS, the slides were analyzed with fluorescence microscopy.

## 3. Results 

### 3.1. Production of Adenovirus Harboring PDX-1, NeuroD1 or MafA

The sequences of the resultant recombinant adenoviruses, which encode PDX-1, NeuroD1, MafA, and GFP, were confirmed by gene sequencing and restriction endonuclease digestions with Pac I. Pac-I-digested adenoviral vectors were transfected into the 293A cell line to produce a crude adenoviral stock and the adenovirus was amplified by infecting 293 A cells. Various kinds of cells can be infected with this adenovirus.

### 3.2. Derivation and Characterization of mMSCs In Vitro

**Figure 1:**
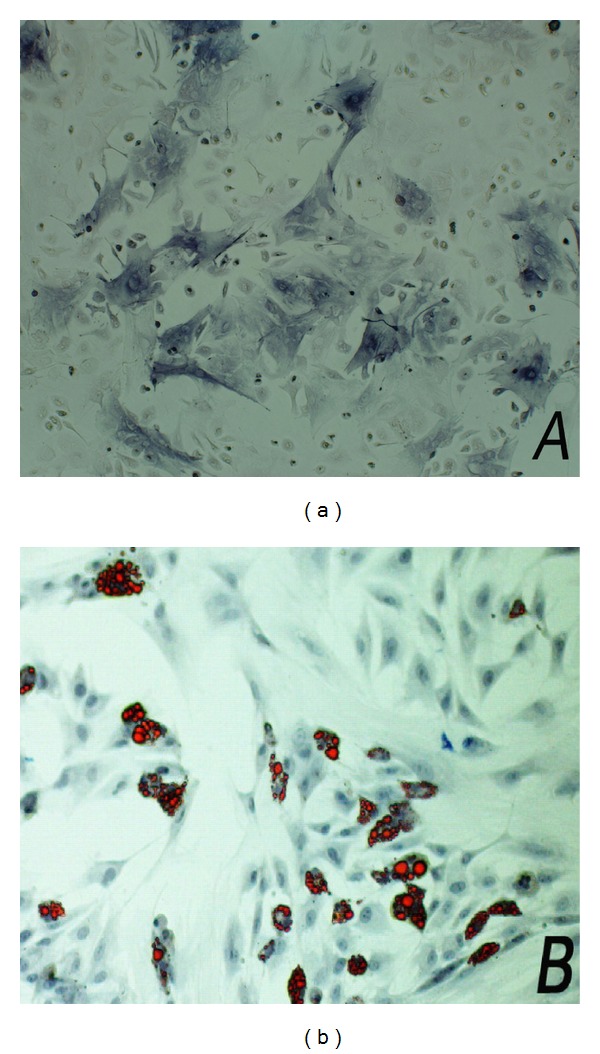
mMSC were induced into osteoblasts and adipocytes in vitro under different differentiation medium. After incubation for 21 days, the differentiated cells were stained with alkaline phosphatase (a) or oil red (b) for their multipotent characteristic.

The unattached cells from the bone marrow samples were removed through medium changes, and the adherent mMSCs were cultured for propagation. After subsequent passaging, most of the adherent cells exhibited fairly uniformly appearance. Immunophenotypes of the cells at passage 3 were assayed by flow cytometry analysis. The majority of the cells expressed high levels of CD29, CD44, and CD105. Meanwhile, the markers CD14, CD34, and CD45 displayed extremely low expression. mMSCs were incubated in osteogenic and adipogenic differentiation medium to identify the multipotency character, and it showed osteogenic differentiation and adipogenic differentiation after 21 days (Figure 1). These results indicated that the cultured cells were in the undifferentiated state and distinguished from haemopoietic stem cells.

### 3.3. Optimizing the MOI of mMSCs

**Figure 2:**
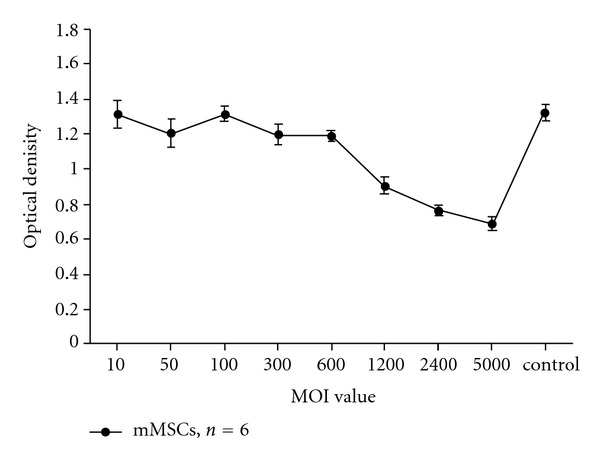
The toxic effect of adenovirus on mMSCs at different multiplicity of infection was determined by CCK-8. 10 *μ*L CCK-8 solution was added into the same amount of mMSCs which were infected with Ad-GFP at different MOI. Optical density of each well varied directly with the survival of the cells. This experiment was repeated six times. Values are mean ± SD.

The susceptibility of different types of cells to adenovirus is variable and differs significantly among the cell types. To achieve the optimal infection with adenovirus, we chose the optimal MOI to raise the infection efficiency and also to have the least amount of cytotoxicity to the mMSCs simultaneously. The results indicated that the infection efficiency improved constantly with the increasing MOI. When cells were infected with freshly adenovirus at MOI of 100, the infection efficiency was over 80%. When cells were infected with fresh adenovirus at an MOI of more than 2,400, the infection rate was almost 100%. On the other hand, the cell survival became significantly inhibited with increasing MOI values beyond 600. The survival curves showed that the number of living infected cells decreased markedly when the MOI value was greater than 600 (Figure 2). From what had been shown above, we conclude that infection of cells at an MOI of 600 is the optimal choice. The cells were infected with viruses containing the three factors at an MOI of 600. mMSCs were infected with diverse single recombinant adenovirus, respectively, at an MOI of 200. mMSCs were infected with viruses containing any two of these factors at an MOI of 400. The infected cells became round in morphology and gave off strikingly bright green fluorescence 3 days after infection. 

### 3.4. Combination of PDX-1, NeuroD1, and MafA Induces IPCs from mMSCs Significantly In Vitro

**Figure 3:**
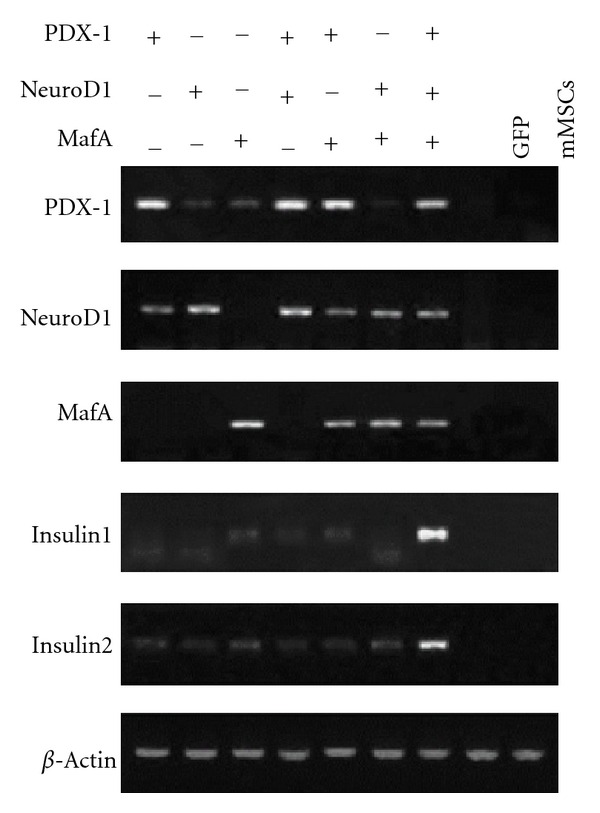
Adenovirus-mediated expression of PDX-1, NeuroD1, and MafA together induced expression of the insulin gene in infected mMSCs. mMSCs were infected with diverse single recombinant adenoviruses, both of the two adenoviruses, a combination of the three adenoviruses, or Ad-GFP. Total RNA from mMSCs was isolated 3 d after infection, and RT-PCR analysis was performed to examine expression of the specified genes. Cultured mMSCs without infection served as the negative control.

**Figure 4:**
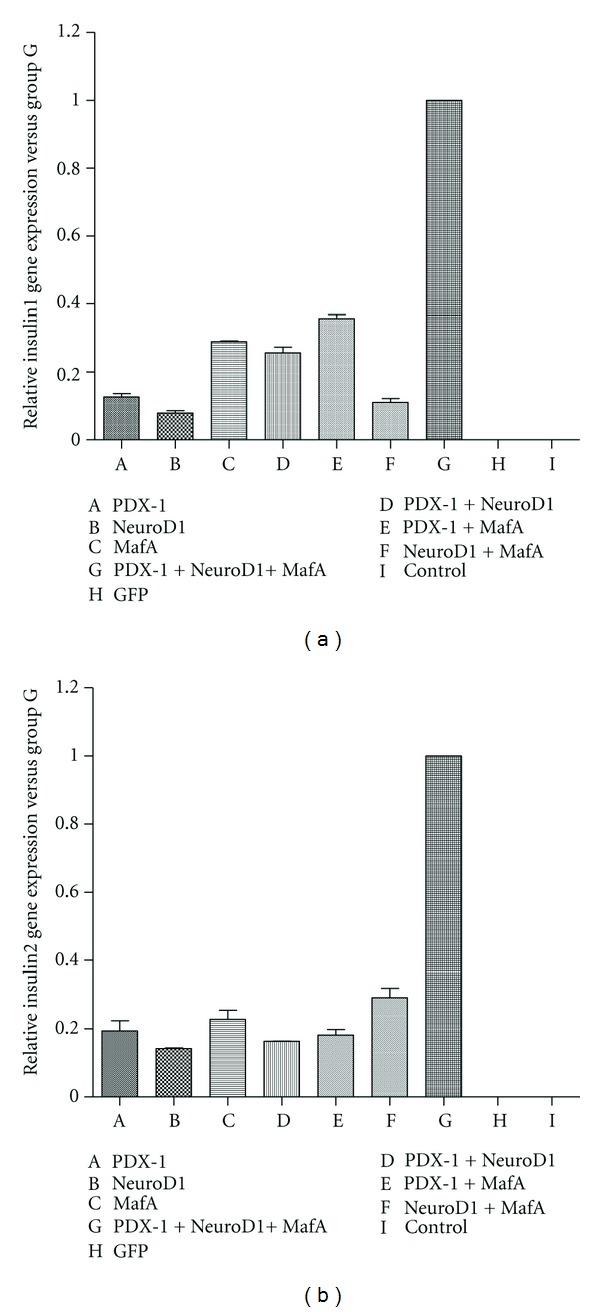
Quantitation of the amount of insulin1 and insulin2 that were produced by the infected mMSCs. The amount of insulin1 (a) and insulin2 (b) mRNA expression in mMSCs with triple infection was significantly larger compared with any other group.

To determine whether the endogenous insulin gene started transcription, gene expression profiles of exogenous transcription factors and insulin gene were evaluated by RT-PCR. As illustrated in Figure 3, the insulin gene and the transcription factor genes were expressed in mMSCs, which were infected with the corresponding transcription factors. The effect of combination of the three factors was more profound compared with any other groups. Consequently, the amount of insulin1 and insulin2 mRNA expression in mMSCs with triple infection was also much larger than any other groups (Figure 4). In contrast, mMSCs infected with Ad-GFP or null, treated with the same culture condition expressed no detectable level of insulin gene or transcription factor gene. It is worthwhile to note that transcription of PDX-1 and NeuroD1 could be mutual activating, and exogenous MafA could trigger the expression of the endogenous PDX-1 in mMSCs.

**Figure 5:**
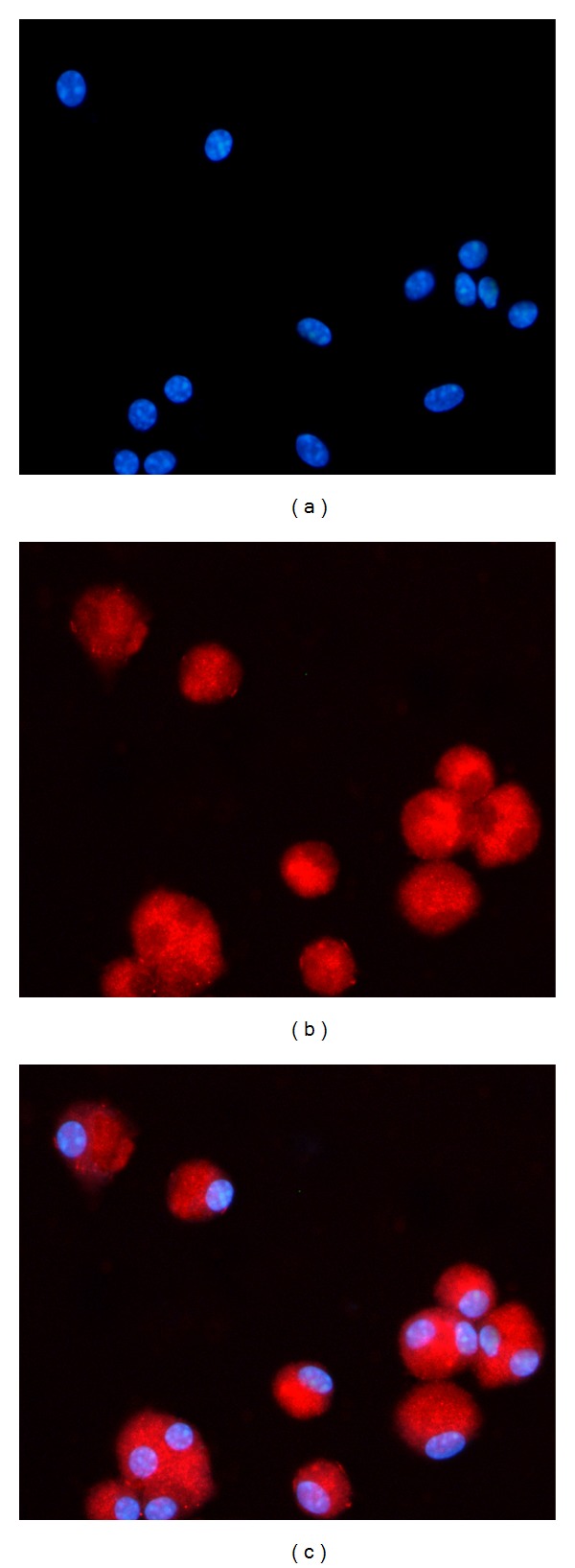
Expression of insulin protein in the infected cells. After culturing them for 21 days, all the infected cells were incubated with anti-mouse insulin antibody. Nuclei were stained blue with Hoechst dye (a). Most of the transgenic cells were stained positively for insulin (b). In contrast, the mMSCs infected with Ad-GFP or null were negative for insulin.

After infection, to determine the biosynthesis of insulin and assay the insulin expression at the protein level, the differentiated mMSCs were documented by immunofluorescence analyses. All the cells on the slides were incubated with anti-mouse insulin and then red fluorescence was clearly visualized in the nucleus and cytoplasm after the triple infection (Figure 5). Nuclei were stained blue with hoechst dye. Red positive reactions were also observed after single factor infection or both of the two factors infection. In contrast, the cultured cells infected with Ad-GFP or null were negative for insulin.

**Figure 6:**
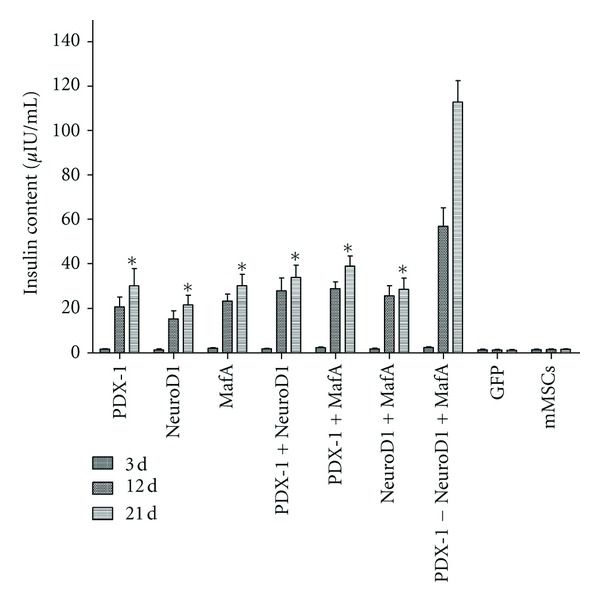
Insulin secretion of the infected cells which were transferred into PDX-1, NeuroD1, MafA, or GFP at different differentiation stages in vitro. The cells were incubated in KRB containing the indicated concentration of glucose. The buffer was then collected for assay of insulin release in each experimental group. One asterisk, *P < 0.05. Data are presented as mean ± SD.

To further determine whether the function of insulin secretion of the differentiated mMSCs, the amount of insulin released by the cells in vitro at indicated concentrations of glucose was measured using a mouse insulin ELISA kit. As illustrated in Figure 6, the results showed that the insulin content of the differentiated mMSCs which were infected with combination of the three transcription factors was significantly higher than that of any other groups (*P* < 0.05). In addition, no insulin release was detected in the buffer added to the cells infected with Ad-GFP or null.

### 3.5. Function Identification of Induced Insulin-Producing Cells In Vivo

**Figure 7:**
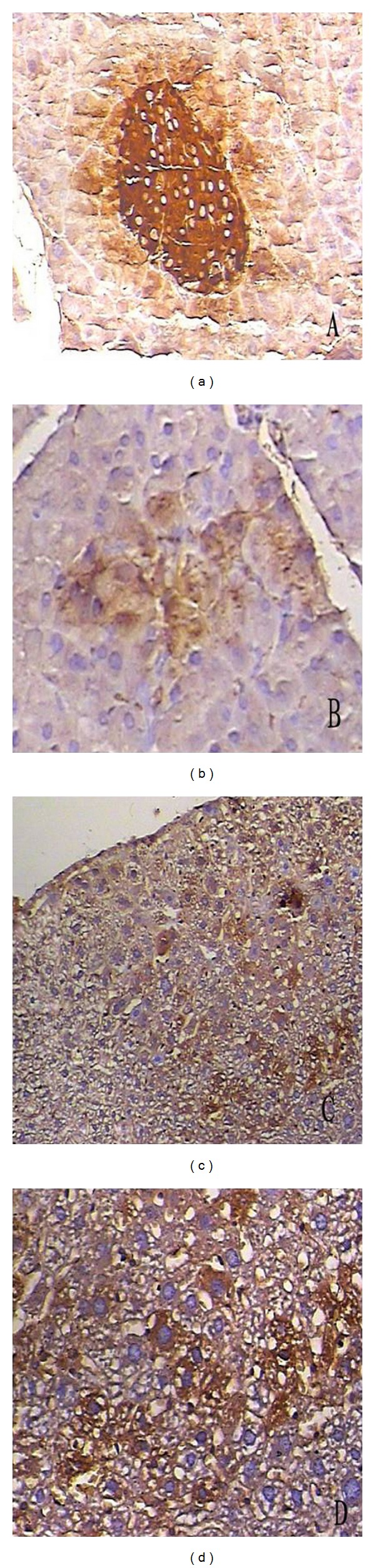
Immunohistochemistry assay for insulin of the survival infected mMSCs in the liver tissues of mice with diabetes. (a) Positive control, anti-mouse insulin staining of mouse pancreatic specimen showing an intense expression of insulin (enlargement ×100). (b) Anti-mouse insulin staining of pancreatic specimen of mice with STZ-induced diabetes showing a markedly decreased expression of insulin (enlargement ×100). (c) Infected mMSCs were injected into the livers of mice with diabetes three days after infection. The positive staining of mouse insulin expression can be clearly observed in the liver. (d) An enlargement of induced IPCs in the liver.

**Figure 8:**
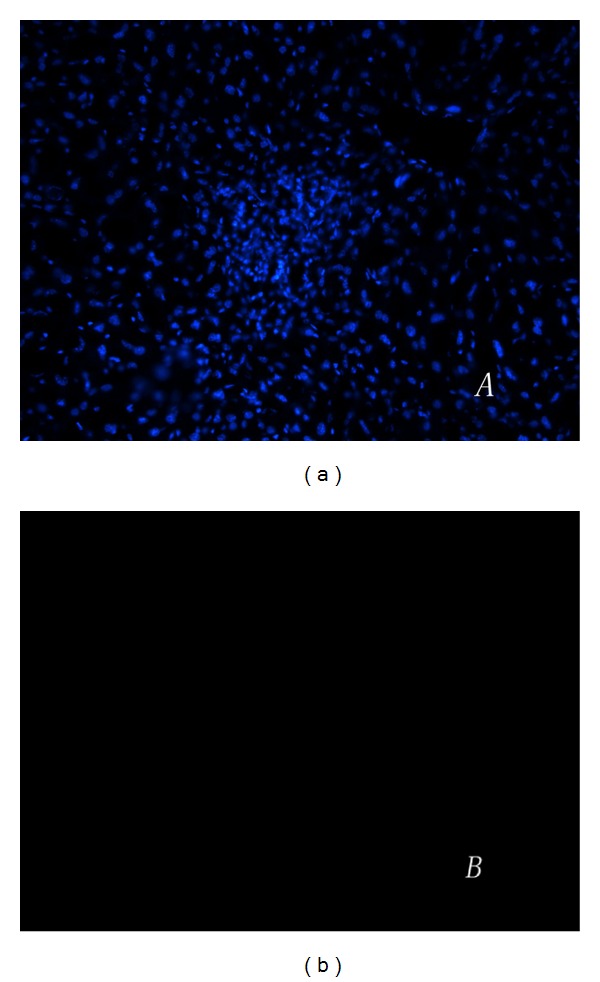
TUNEL assay was performed to see whether the injected cells were apoptotic. (a) Frozen tissue sections of the livers were stained with hoechst. (b) The immunofluorescent stainings of TUNEL were negative in the transplanted cells.

**Figure 9:**
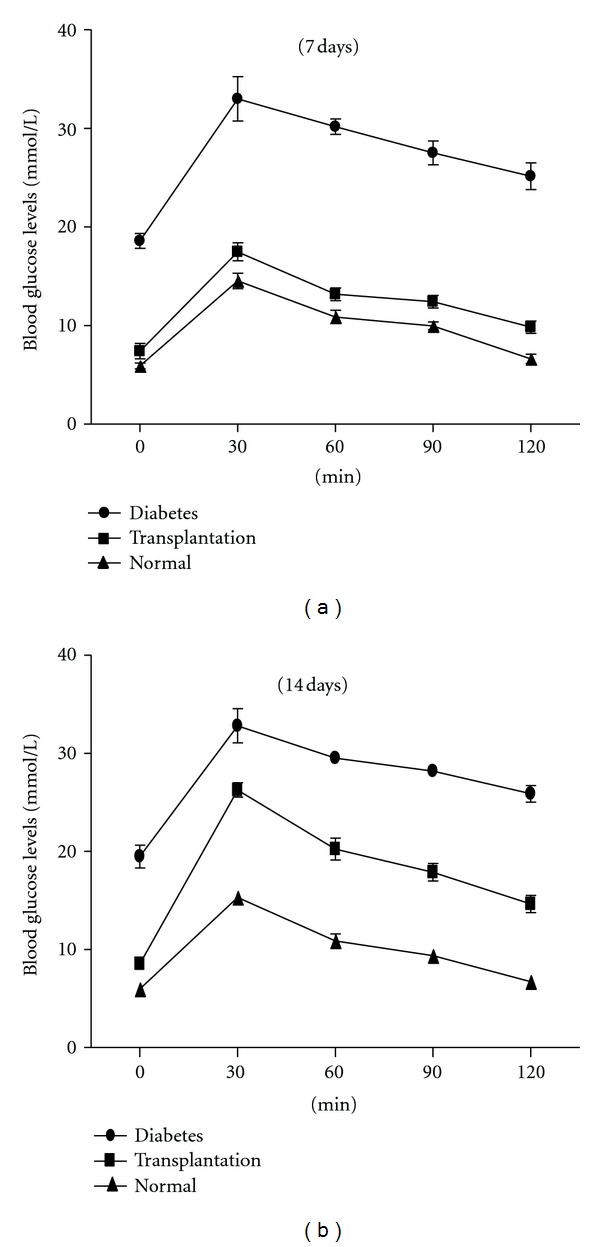
Glucose responses to glucose tolerance test of mice with diabetes after transplantation. The infected cells which expressed combination of PDX-1, NeuroD1, and MafA were transplanted into the livers of mice with STZ-induced diabetes, glucose tolerance test was performed at 7 (a) and 14 days (b) following transplantation, compared with a normal control and diabetes models without any treatment. Data are presented as mean ± SD.

Take a step further to determine whether the induced IPCs give full scope to normal physiological functions of *β* islet cells, mMSCs expressing a combination of PDX-1, NeuroD1, and MafA were transplanted into the livers of mice with STZ-induced diabetes. mMSCs infected with Ad-GFP or without any infection were transplanted into the livers as a control. First, we examined insulin protein expression and cell apoptosis in the tissue of liver. Insulin content was not detected in the liver of mice treated with mMSCs without infection but was indeed clearly detected after treatment with mMSCs expressed combination of PDX-1, NeuroD1, and MafA (Figure 7). The immunofluorescent stainings of TUNEL were negative in the injected cells (Figure 8), which indicated that they had never experienced double-strand DNA breaks associated with apoptosis. In addition, insulin protein expression was substantially diminished after 1 month and was not detectable after 2 months. Furthermore, to assess the contribution on controlling blood glucose levels of insulin produced by the engrafted cells, a glucose tolerance test was performed 7–14 days after transplantation. As shown in Figure 9, the result revealed that mMSCs expressing a combination of PDX-1, NeuroD1, and MafA were able to respond to the glucose challenge, and their response was almost comparable to that of normal *β*-islet cells 7 days after transplantation. Notably, the same or better effect was not elicited after another 7 days. This is probably due to the fact that unstable and transient transgene expression in the cells, plus induced cells failed to materialize self-reproduction. There were no differences in blood glucose levels at any time point between mice with STZ-induced diabetes implanted with normal mMSCs and nontransplantation.

## 4. Discussion

In recent years, cell transplantation has become a research hotspot concerning surgical methods for the treatment of diabetes. In order to obtain surrogate *β*-cells, the target cells were transdifferentiated, dedifferentiated, or differentiated to surrogate *β*-cells in the usual by expressing some key transcription factors involved in the pancreas development and *β*-cell gene expression [18, 19]. In this study we report a procedure for delivery of combination of PDX-1, NeuroD1, and MafA into mMSCs on adenoviral vectors and their differentiation into IPCs in vitro or in vivo. Our results demonstrate that bone marrow mesenchymal stem cells were induced for directional differentiation into IPCs by a combination of just three key transcription factors. Overexpression of PDX-1, NeuroD1, and MafA markedly upregulated the expression of insulin gene and also induced insulin biosynthesis and secretion in mMSCs. The triple infection has a much stronger influence compared with any single or double infection. 

The *β* islet cells are unique in their ability to produce, process, and secrete significant amounts of insulin in a strictly regulated manner in response to continuously varying concentrations of glucose [20]. The development process and function maintenance of *β*-cells demand networking regulation consisting of several transcription factors. 

Previous research has suggested that stable expression of PDX-1 in adult human mesodermal tissues activated expression of all four islet hormones including insulin and reversed hyperglycemia in vivo, but more factors that stimulate cells further toward differentiated normal *β*-cells were needed [10]. In our study, any single factor and combinations of any two factors were able to induce expression of insulin, but the effect elicited in mMSCs was too weak relative to the particular combination of these three factors. It is apparently not sufficient to drive differentiation of mMSCs a long way toward *β*-cells or IPCs in the treatment of diabetes. A certain fact to be reckoned with is that all the three transcription factors are bound to the A3, E1, and C1 sites in a 340 bp promoter region upstream of the transcription start site of the insulin gene [21–25]. In contrast or for further research, we developed our experiments in vivo so that induced IPCs would reside in their native environment and might be promoted in their survival and maturation. As the homologous feature between the liver and the pancreas has been displayed in many animal samples [26], transplantation experiments and in vitro differentiation experiments [27], in addition that the liver is the primary organ where insulin functions, we think the liver tissue is an ideal microenvironment for IPCs to survive and function. Further work will be to explore if additional factors are necessary for the particular combination and mechanism among actions of the factors.

In the experiments of gene detection, genetic transformation of PDX-1 activated the expression of endogeneous NeuroD1 and endogeneous PDX-1 could be activated by exogenous NeuroD1 or MafA. The experimental results indicated that adjustment or interaction may really exist between each transcription factor. However, PDX-1 and MafA, together with endogeneous NeuroD1 were unable to exert as strong an influence on the expression of the insulin gene as delivery of a combination of the three transcription factors. We assume that fine synergism could not be achieved due to the low expression level of induced factors. 

Intracellular GFP of the mMSCs was subsequently initiated to expression at 3 days after gene delivery, close together with the factors. However, one week, later, the intensity of the fluorescence decreased with the degradation of partial mitochondrial DNA. Therefore, induced efficiency was significantly inhibited without a repetition of infection. Cell transplantation in liver parenchyma was done to further verify the function of induced IPCs in vivo. Both intraperitoneal injection and high carbohydrate feeding are the methods recommended by researchers for glucose tolerance test. Comparatively, intraperitoneal injection goes in a more accurate way for mice and is also simple to perform. The results of an IPGTT demonstrated the ability of these implanted cells to dispose of a glucose load, and the glucose tolerance was close to normal mice. However, it should be noted that impaired glucose tolerance was found after another 7 days. It may be the case that the implanted induced IPCs failed to proliferate. Strategies that make stable expression of the factors in mMSCs may possibly help to evaluate the long term effect of the treatment. 

Bone marrow mesenchymal stem cell has been known for their multiplex differentiation potential and relative ease to obtain. They were able to be modified to develop epigenetic changes, which were controlled by a series of several distinct related genes, and then differentiated into functional *β*-cells. 

In conclusion, our findings demonstrated that genetic manipulation producing infection by a combination of PDX-1 NeuroD1, and MafA and their subsequent expression significantly promoted insulin-producing function of mMSCs. Although substantial work has been done, the effective approach related to generation of surrogate *β*-cells for the treatment of diabetes is still not obtained. Nevertheless, we will further identify the differentiation of mMSCs expressed combination of the just three factors in vivo and their stable long-term expression for maintaining strict blood glucose levels.

## References

[1] Best M, Carroll M, Hanley NA, Piper Hanley K (2008). Embryonic stem cells to *β*-cells by understanding pancreas development. *Molecular and Cellular Endocrinology*.

[2] Kim JH, Shin KH, Li TZ, Suh H (2011). Potential of nucleofected human MSCs for insulin secretion. *Journal of Tissue Engineering and Regenerative Medicine*.

[3] Li G, Luo R, Zhang J (2009). Generating mESC-derived insulin-producing cell lines through an intermediate lineage-restricted progenitor line. *Stem Cell Research*.

[4] Naujok O, Francini F, Picton S, Bailey CJ, Lenzen S, Jörns A (2009). Changes in gene expression and morphology of mouse embryonic stem cells on differentiation into insulin-producing cells in vitro and in vivo. *Diabetes/Metabolism Research and Reviews*.

[5] Bernardo AS, Hay CW, Docherty K (2008). Pancreatic transcription factors and their role in the birth, life and survival of the pancreatic *β* cell. *Molecular and Cellular Endocrinology*.

[6] Andrali SS, Smapley ML, Vanderford NL, Ozcan S (2008). Glucose regulation of insulin gene expression in pancreatic *β*-cells. *Biochemical Journal*.

[7] Karaoz E, Aksoy A, Ayhan S, SarIboyacI AE, Kaymaz F, Kasap M (2009). Characterization of mesenchymal stem cells from rat bone marrow: ultrastructural properties, differentiation potential and immunophenotypic markers. *Histochemistry and Cell Biology*.

[8] Tonti GA, Mannello F (2008). From bone marrow to therapeutic applications: different behaviour and genetic/epigenetic stability during mesenchymal stem cell expansion in autologous and foetal bovine sera?. *International Journal of Developmental Biology*.

[9] Zhao S, Wehner R, Bornhauser M, Wassmuth R, Bachmann M, Schmitz M (2010). Immunomodulatory properties of mesenchymal stromal cells and their therapeutic consequences for immune-mediated disorders. *Stem Cells and Development*.

[10] Karnieli O, Izhar-Prato Y, Bulvik S, Efrat S (2007). Generation of insulin-producing cells from human bone marrow mesenchymal stem cells by genetic manipulation. *Stem Cells*.

[11] Li Y, Zhang R, Qiao H (2007). Generation of insulin-producing cells from PDX-1 gene-modified human mesenchymal stem cells. *Journal of Cellular Physiology*.

[12] Lu Y, Wang Z, Zhu M (2006). Human bone marrow mesenchymal stem cells transfected with human insulin genes can secrete insulin stably. *Annals of Clinical and Laboratory Science*.

[13] Xu J, Lu Y, Ding F, Zhan X, Zhu M, Wang Z (2007). Reversal of diabetes in mice by intrahepatic injection of bone-derived GFP-murine mesenchymal stem cells infected with the recombinant retrovirus-carrying human insulin gene. *World Journal of Surgery*.

[14] Kim J, Lengner CJ, Kirak O (2011). Reprogramming of postnatal neurons into induced pluripotent stem cells by defined factors. *Stem Cells*.

[15] Ambasudhan R, Talantova M, Coleman R (2011). Direct reprogramming of adult human fibroblasts to functional neurons under defined conditions. *Cell Stem Cell*.

[16] Guo Z, Li H, Li X (2006). In vitro characteristics and in vivo immunosuppressive activity of compact bone-derived murine mesenchymal progenitor cells. *Stem Cells*.

[17] Maxson S, Burg KJL (2008). Conditioned media cause increases in select osteogenic and adipogenic differentiation markers in mesenchymal stem cell cultures. *Journal of Tissue Engineering and Regenerative Medicine*.

[18] Minami K, Seino S (2008). Pancreatic acinar-to-beta cell transdifferentiation in vitro. *Frontiers in Bioscience*.

[19] Meivar-Levy I, Ferber S (2010). Adult cell fate reprogramming: converting liver to pancreas. *Methods in Molecular Biology*.

[20] Miller K, Kim A, Kilimnik G (2009). Islet formation during the neonatal development in mice. *PLoS ONE*.

[21] Hay CW, Docherty K (2006). Comparative analysis of insulin gene promoters: implications for diabetes research. *Diabetes*.

[22] Peshavaria M, Cissell MA, Henderson E, Petersen HV, Stein R (2000). The PDX-1 activation domain provides specific functions necessary for transcriptional stimulation in pancreatic *β*-cells. *Molecular Endocrinology*.

[23] Kaneto H, Matsuoka TA, Kawashima S (2009). Role of MafA in pancreatic *β*-cells. *Advanced Drug Delivery Reviews*.

[24] Hagman DK, Hays LB, Parazzoli SD, Poitout V (2005). Palmitate inhibits insulin gene expression by altering PDX-1 nuclear localization and reducing MafA expression in isolated rat islets of Langerhans. *The Journal of Biological Chemistry*.

[25] Aramata S, Han SI, Yasuda K, Kataoka K (2005). Synergistic activation of the insulin gene promoter by the *β*-cell enriched transcription factors MafA, Beta2, and Pdx1. *Biochimica et Biophysica Acta*.

[26] Jung J, Zheng M, Goldfarb M, Zaret KS (1999). Initiation of mammalian liver development from endoderm by fibroblast growth factors. *Science*.

[27] Suzuki A, Zheng YW, Kaneko S (2002). Clonal identification and characterization of self-renewing pluripotent stem cells in the developing liver. *Journal of Cell Biology*.

